# Study of the synergistic anti-inflammatory activity of *Solanum xanthocarpum* Schrad and Wendl and *Cassia fistula* Linn

**DOI:** 10.4103/0974-7788.72489

**Published:** 2010

**Authors:** Shraddha Anwikar, Milind Bhitre

**Affiliations:** *C. U. Shah College of Pharmacy, SNDT Women’s University, Santacruz (W), Mumbai, India*

**Keywords:** Anti-inflammatory activity, *Cassia fistula* linn, isobolographic analysis, *Solanum xanthocarpum* Schrad and Wendl

## Abstract

**Introduction:**

Nonsteroidal and steroidal drugs are generally used as a part of drug therapy in inflammation. However, these drugs have severe side-effects like nausea and vomiting. Therefore, there is a need to identify anti-inflammatory compounds that will be effective with a better safety profile. Solanum xanthocarpum Schrad and Wendl and Cassia fistula Linn has many therapeutic uses mentioned in Ayurveda and therefore we aimed to study its anti-inflammatory activity both alone and in combination.

**Materials and Methods:**

The water extract of dried fruits of Solanum xanthocarpum Schrad and Wendl and dried pulp of Cassia fistula Linn was prepared. The anti-inflammatory activity of these extracts was investigated using the carragenan-induced paw edema model in rats individually and in two different combinations. ED50 of both the extracts singly and in combination were calculated by dose-response curves, and this information was then plotted on the isobologram. The interaction index of the extracts was also investigated to determine whether both the extracts in combination show synergistic or antagonistic or additive effects.

**Results:**

It was observed that extracts of dried fruits of Solanum xanthocarpum showed more anti-inflammatory activity than dried fruits of Cassia fistula Linn. Both the extracts showed maximum anti-inflammatory activity at 500 mg/kg dose. Among the different dose combinations of both the extracts, the 1:1 combination at the 500 mg/kg dose showed maximum percentage inhibition of 75%, which was comparable with the positive control, diclofenac sodium, which showed 81% inhibition.

**Conclusion:**

As revealed by the isobolograms, both the combinations fell below the additivity line, which indicates synergistic interactions between Solanum xanthocarpum and Cassia fistula extracts. Interaction indices of both combinations were observed to be <1, which re-demonstrated the synergistic effects of the combination.

## INTRODUCTION

Inflammation is the body defense reaction to eliminate or limit the spread of an injurious agent as well as to remove the consequently necrosed cells and tissues. Although inflammation is often undesirable, because an inflamed throat, skin or soft tissue can cause considerable discomfort, it is actually a beneficial and defensive phenomenon. The net result is the neutralization and elimination of an offending agent, the demolition of necrotic tissue and the establishment of conditions necessary for repair and restitution.[1]

Nonsteroidal and steroidal drugs are generally used to treat inflammation. However, these drugs have side-effects like nausea, vomiting, etc.[2] This led us to search for new anti-inflammatory agents from natural sources, which would be effective and safe. Many plant extracts show a synergistic effect with each other or with modern drugs. The extracts of *Solanum xanthocarpum* Schrad and Wendl and *Cassia fistula* Linn have been reported to possess anti-inflammatory activity.[3,4] We conducted this study to determine whether both these plant extracts showed increased anti-inflammatory activity at low dose when given in combination.

## MATERIALS AND METHODS

### Procurement and authentication of plant samples

Dried fruits of *Solanum xanthocarpum* and fruit pulp of *Cassia fistula* were procured from the local market in Mumbai and were authenticated at Nicholas Piramal Life Sciences, Mumbai. Fruits were powdered with a mechanical grinder and stored in an airtight container. The powdered drugs were tested for routine quality parameters like total ash, moisture content, sulfated ash, insoluble ash, etc.[5]

### Preparation of plant extract[6]

Aqueous extraction of the powdered dried fruits of both the plants was carried out separately in a Soxhelet apparatus for 18 h. The extract was dried to a constant weight and kept under refrigeration. The extract obtained was standardized for parameters like %yield, color and consistency and used for further study.

### Phytochemical screening of the extracts

Both the extracts were subjected to various tests to check the phytochemical constituents, viz. carbohydrates, glycosides, tannins, alkaloids, saponins, inorganic matter, etc.[5]

### Anti-inflammatory study[7–10]

Wistar Albino rats of either sex with body weight 150–300 g were used for the study. The animals were housed in cages under standard laboratory conditions (12-h light/dark cycle at 25° ± 2°C). They were allowed free access to a standard dry pellet diet (Hindustan Lever Ltd. Mumbai, India) and water *ad libitum*. All procedures described were reviewed and approved by the SNDT Women’s University Animal Ethics Committee.

Animals were fasted overnight before administering the study drugs. Animals were divided into a total of six groups, as positive control, negative control, *Solanum xanthocarpum* group, *Cassia fistula* group, combination 1 and combination 2.

Diclofenac sodium at a dose of 150 mg/kg was administered to the positive control group animals. Oral single doses of individual plant extracts ranged from 100 to 500 mg/kg and a combination of both plant extracts were administered to the other group of animals, as shown in Table 1.

**Table 1:** Doses administered to animals

Group 1:	Positive control
Group 2:	Negative control
Group 3:	*Solanum xanthocarpum* extract
	Subgroup 1: 6 animals administered 100 mg/kg *Solanum xanthocarpum* extract
	Subgroup 2: 6 animals administered 200 mg/kg *Solanum xanthocarpum* extract
	Subgroup 3: 6 animals administered 300 mg/kg *Solanum xanthocarpum* extract
	Subgroup 4: 6 animals administered 400 mg/kg *Solanum xanthocarpum* extract
	Subgroup 5: 6 animals administered 500 mg/kg *Solanum xanthocarpum* extract
Group 4:	*Cassia fistula* extract
	Subgroup 1: 6 animals administered 100 mg/kg *Cassia fistula* extract
	Subgroup 2: 6 animals administered 200 mg/kg *Cassia fistula* extract
	Subgroup 3: 6 animals administered 300 mg/kg *Cassia fistula* extract
	Subgroup 4: 6 animals administered 400 mg/kg *Cassia fistula* extract
	Subgroup 5: 6 animals administered 500 mg/kg *Cassia fistula* extract
Group 4:	Combination 1
	Subgroup 1: 6 animals administered 250 mg/kg SX extract and 50 mg CF extract
	Subgroup 2: 6 animals administered 250 mg/kg SX extract and 100 mg CF extract
	Subgroup 3: 6 animals administered 250 mg/kg SX extract and 150 mg CF extract
	Subgroup 4: 6 animals administered 250 mg/kg SX extract and 250 mg CF extract
Group 4:	Combination 2
	Subgroup 1: 6 animals administered 250 mg/kg CF extract and 50 mg SX extract
	Subgroup 2: 6 animals administered 250 mg/kg CF extract and 100 mg SX extract
	Subgroup 3: 6 animals administered 250 mg/kg CF extract and 150 mg SX extract

SX: *Solanum xanthocarpum*, CF: *Cassia fistula*

After 30 min of the dose administration as shown in Table 1, rats were challenged by subcutaneous injection of 0.1 ml of 1% solution of carrageenen into the planter side of the right hind paw. The paw volume was measured plethysmographically immediately after injection and again 1, 2, 3 and 4 h after challenge.

The increase of paw volume from 0 to 4 h was calculated as a percentage. The difference of average values between treated animals and control groups was calculated for each time interval and compared statistically.

The percentage inhibition was calculated according to the following formula:[9]

Percentage inhibition = 100 (1 - [A - X/B - Y])

where, A- mean paw volume of treated group after carrageenan injection.

B- mean paw volume of control group after carrageenan injection.

X- mean paw volume of treated group before carrageenan injection.

Y- mean paw volume of control group before carrageenan injection.

### Isobolographic analysis of the response

The assessment of synergism is most often made from experiments in which an effect level is chosen and doses of drug A alone, drug B alone and the combination (a,b) that give this effect are determined experimentally. Doses that give the same effect are called isoboles and the method of analysis is an isobolographic method.

A measure of the synergism is made from the value of the interaction index. The index, denoted by γ, is defined by the isobolar relation.

Interaction index: a/A + b/B = γ

where, A = ED50 of *Solanum xanthocarpum* Schrad and Wendl

B = ED50 of *Cassia fistula* Linn

(a,b)= Dose of combination that shows ED50

The quantities in the above equation are obtained from the dose-response curves of drugs A, B and the combination.

If γ = 1, the interaction is additive,

if γ <1, the interaction is synergistic and

if γ >1, the interaction is antagonistic in nature.

Isobologram is plotted between dose of drug A and drug B. Doses that give a specified effect are joined, which is called as additivity line. Combinations are plotted on an isobologram: if that combination lies below the additivity line, it shows a synergistic activity and if it lies on the additivity line, it shows an additive activity. If it is beyond the additivity line, an antagonistic activity is indicated.[11]

## RESULTS

### Phytochemical screening

The phytochemical analysis revealed the presence of carbohydrates, amino acids, proteins and anthraquinone glycosides in the *Cassia fistula* extract and carbohydrates, saponins, steroids, alkaloids, amino acids and proteins in the *Solanum xanthocarpum* extracts.

### Anti-inflammatory study

**Figure 1:**
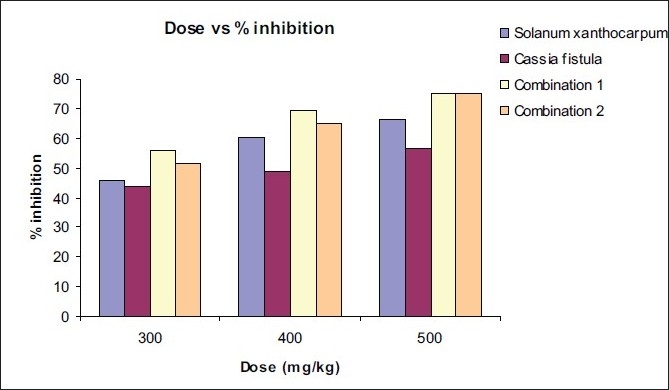
Percentage inhibition of both plant extracts and the combination at the 300, 400 and 500 mg/kg doses

The effects of both the extracts alone and in combination on paw edema induced by carrageenan are shown in Table 2. Percentage inhibition of both the extracts and the combination at 300, 400 and 500 mg/kg dose is shown in Table 3 [Figure 1].

### Isobolographic analysis

**Figure 2:**
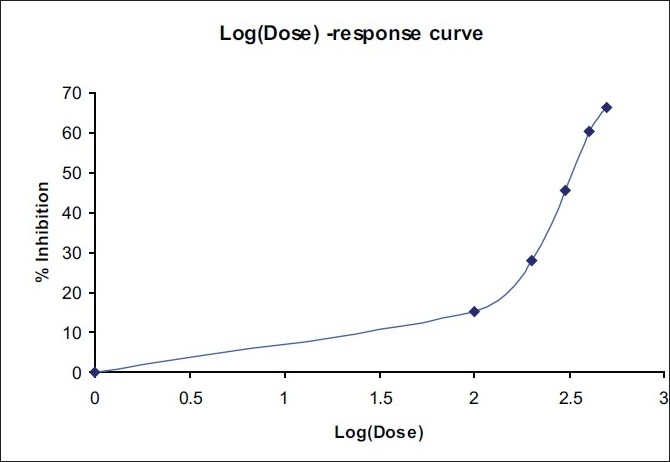
Dose response curve of *Solanum xanthocarpum* Schrad and Wendl

**Figure 3:**
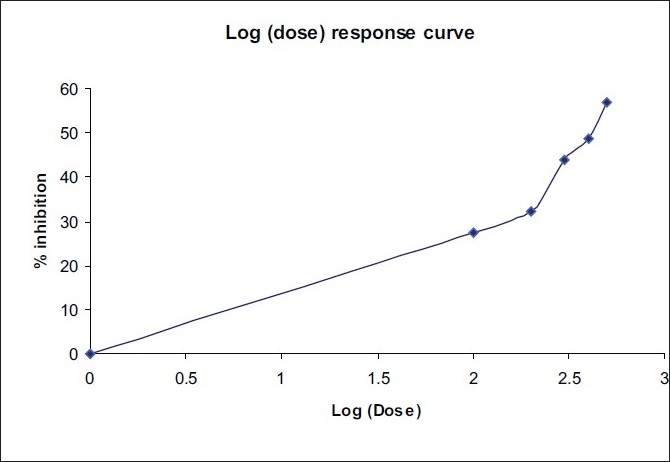
Dose-response curve of *Cassia fistula* Linn

**Figure 4:**
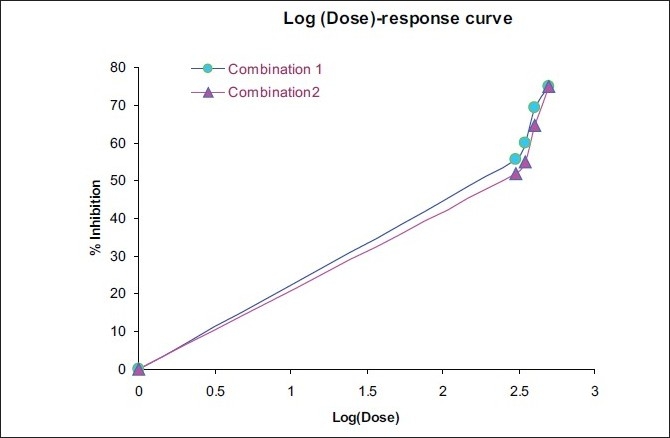
Dose-response curve of combination 1 and combination 2

ED50 of both the plant extracts alone and at fixed ratios was assessed by an isobolographic analysis of the dose-response curves [Table 4; Figures 2–4]. The interaction index was found to be 0.64 for combination 1 and 0.88 for combination 2, which shows a synergistic activity.

**Table 2:** Percentage inhibition of *Solanum xanthocarpum* and *Cassia fistula* Linn alone and in combination at different doses

Treatment	Doses (mg/kg)	Average paw volume of rats					Mean difference in fourth-hour paw volume (X)		Percentage inhibition
		0 h	1^st^ h	2^nd^ h	3^rd^ h	4^th^ h	X ± SEM	X ± SD	
Positive control		1.11	1.70	2.44	2.73	2.45	1.34 ± 0.102	1.34 ± 0.249	81
Negative control		1.61	1.62	1.85	1.98	1.87	0.26 ± 0.063	0.26 ± 0.154	
Solanum xanthocarpum	100	1.11	1.56	1.91	1.64	2.24	1.13 ± 0.099	1.13 ± 0.242	15.11
	200	0.85	1.51	1.79	1.89	1.82	0.97 ± 0.049	0.97 ± 0.120	27.62
	300	1.05	1.49	1.97	2.09	1.77	0.72 ± 0.051	0.72 ± 0.125	45.65
	400	1.11	1.56	1.91	1.77	1.64	0.53 ± 0.035	0.53 ± 0.086	60.44
	500	1.03	1.58	1.76	1.80	1.48	0.45 ± 0.026	0.45 ± 0.064	66.41
Cassia fistula	100	1.56	2.09	2.56	2.10	2.53	0.97 ± 0.10	0.97 ± 0.245	27.6
	200	1.48	2.09	2.49	2.40	2.38	0.90 ± 0.097	0.90 ± 0.238	32.26
	300	1.67	2.21	2.34	2.46	2.42	0.75 ± 0.095	0.75 ± 0.233	43.81
	400	1.43	1.73	2.23	2.20	2.11	0.68 ± 0.089	0.68 ± 0.218	48.74
	500	0.97	1.40	1.69	1.84	1.55	0.58 ± 0.077	0.58 ± 0.189	56.90
Combination 1	250 SX +50CF	1.67	1.86	1.96	2.28	2.26	0.59 ± 0.080	0.59 ± 0.196	55.72
	250SX + 100CF	1.19	1.59	1.99	2.11	1.72	0.53 ± 0.072	0.53 ± 0.176	60.11
	250SX + 150CF	1.56	1.72	1.93	2.03	1.97	0.41 ± 0.056	0.41 ± 0.137	69.49
	250SX + 250CF	1.15	1.22	1.39	1.50	1.48	0.33 ± 0.038	0.33 ± 0.093	75
Combination 2	250 CF+50SX	0.93	1.15	1.81	1.85	1.58	0.65 ± 0.079	0.65 ± 0.193	51.77
	250 CF+100SX	0.97	1.60	1.63	1.72	1.57	0.60 ± 0.041	0.60 ± 0.1	54.88
	250 CF+150SX	1.41	1.87	2.09	1.48	1.88	0.47 ± 0.053	0.47 ± 0.13	64.79

**Table 3:** % inhibition of Solanum xanthocarpum Schrad and Wendl and *Cassia fistula* Linn and of both combinations at 300, 400 and 500 mg/kg doses

Plant extract	% inhibition at the dose		
	300 mg/kg	400 mg/kg	500 mg/kg
Solanum xanthocarpum	45.65	60.44	66.41
Cassia fistula	43.81	48.74	56.90
Combination 1	55.72	69.49	75
Combination 2	51.77	64.79	75

**Table 4:** ED50 of *Solanum xanthocarpum* and *Cassia fistula* extracts alone and in combination

Plant extract	Equation	ED_50_ (mg/kg)
Solanum xanthocarpum	Y = 76.727 X- 142.27	320.55
Cassia fistula	Y = 41.651 X- 58.76	408.52
Combination 1	Y = 91.233 X- 170.35	260.16
Combination 2	Y = 109.96 X- 222.15	298.53

## DISCUSSION

It was observed that extracts of *Solanum xanthocarpum* showed a higher anti-inflammatory activity than the extracts of *Cassia fistula* Linn. Both the extracts showed a maximum anti-inflammatory activity at the 500 mg/kg dose. Among different combinations of both the extracts, the 1:1 combination at the 500 mg/kg dose showed the maximum percentage inhibition of 75%, which was comparable with the positive standard diclofenac sodium that showed 81% inhibition.[10]

**Figure 5:**
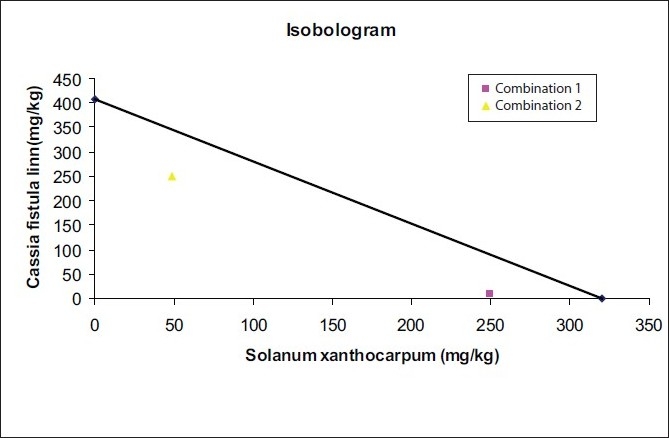
Isobolographic plot for anti-inflammatory effect of *Solanum xanthocarpum* and *Cassia fistula* in combination

Synergistic effect was proved by isobolographic analysis. The doses of both the combinations showing a 50% effect were plotted on the isobolograms. The isobolograms as shown in Figure 5 indicate that both the combinations fall below the additivity line, which shows that synergistic interactions occurred between *Solanum xanthocarpum* and *Cassia fistula* extract.

The interaction index of both the combinations was calculated, which found to be <1. This indicated that both the extracts in combination show a better anti-inflammatory activity than when alone, which confirms the synergistic activity.

To conclude, percentage inhibition of both the extracts in combinations is higher than the individual plant extracts. The interaction index was also observed to be <1. This showed that both the plant extracts were synergistic in nature.
